# Patients’ Expectations Regarding Medical Treatment: A Critical Review of Concepts and Their Assessment

**DOI:** 10.3389/fpsyg.2017.00233

**Published:** 2017-02-21

**Authors:** Johannes A. C. Laferton, Tobias Kube, Stefan Salzmann, Charlotte J. Auer, Meike C. Shedden-Mora

**Affiliations:** ^1^Department of Psychology, Clinical Psychology and Psychotherapy, Psychologische Hochschule BerlinBerlin, Germany; ^2^Department of Psychology, Division of Clinical Psychology and Psychotherapy, Philipps University of MarburgMarburg, Germany; ^3^Division of Psychotherapy and Psychiatry, University Hospital LübeckLübeck, Germany; ^4^Department of Psychosomatic Medicine and Psychotherapy, University Medical Center Hamburg-EppendorfHamburg, Germany

**Keywords:** expectations, outcome expectancy, self-efficacy, optimism, placebo effect, treatment, assessment, operationalization

## Abstract

Patients’ expectations in the context of medical treatment represent a growing area of research, with accumulating evidence suggesting their influence on health outcomes across a variety of medical conditions. However, the aggregation of evidence is complicated due to an inconsistent and disintegrated application of expectation constructs and the heterogeneity of assessment strategies. Therefore, based on current expectation concepts, this critical review provides an integrated model of patients’ expectations in medical treatment. Moreover, we review existing assessment tools in the context of the integrative model of expectations and provide recommendations for improving future assessment. The integrative model includes expectations regarding treatment and patients’ treatment-related behavior. Treatment and behavior outcome expectations can relate to aspects regarding benefits and side effects and can refer to internal (e.g., symptoms) and external outcomes (e.g., reactions of others). Furthermore, timeline, structural and process expectations are important aspects with respect to medical treatment. Additionally, generalized expectations such as generalized self-efficacy or optimism have to be considered. Several instruments assessing different aspects of expectations in medical treatment can be found in the literature. However, many were developed without conceptual standardization and psychometric evaluation. Moreover, they merely assess single aspects of expectations, thus impeding the integration of evidence regarding the differential aspects of expectations. As many instruments assess treatment-specific expectations, they are not comparable between different conditions. To generate a more comprehensive understanding of expectation effects in medical treatments, we recommend that future research should apply standardized, psychometrically evaluated measures, assessing multidimensional aspects of patients’ expectations that are applicable across various medical treatments. In the future, more research is needed on the interrelation of different expectation concepts as well as on factors influencing patients’ expectations of illness and treatment. Considering the importance of patients’ expectations for health outcomes across many medical conditions, an integrated understanding and assessment of such expectations might facilitate interventions aiming to optimize patients’ expectations in order to improve health outcomes.

## Introduction

The relevance of patients’ expectations for health outcomes has received increasing attention in recent years. Expectations play an important role in both physical (Di Blasi et al., 2001; Mondloch et al., 2001) and mental health (Constantino et al., 2011; Rief et al., 2015; Kube et al., 2017). Moreover, they are a key mechanism of the placebo and nocebo effect, a phenomenon according to which subjective and physiological changes emerge due to inert or non-specific treatment components (Colloca and Miller, 2011b; Enck et al., 2013). Accumulating evidence suggests that expectations influence treatment outcome in patients with various medical conditions. For instance, they have been linked to course and treatment outcome in patients with heart disease (Petrie et al., 1996; Juergens et al., 2010; Barefoot et al., 2011; Habibovic et al., 2014), stroke (Jones and Riazi, 2011), cancer (Colagiuri and Zachariae, 2010; Nestoriuc et al., 2016), musculoskeletal disorders (Mahomed et al., 2002; Oettingen and Mayer, 2002; van den Akker-Scheek et al., 2007), injuries (Booth-Kewley et al., 2014; Murgatroyd et al., 2016) and obesity (Oettingen and Wadden, 1991; Armitage et al., 2015; Crane et al., 2016). Expectations even predict outcome in patients undergoing different kinds of surgery (Auer et al., 2016a). Hence, patients with more positive expectations seem to be more likely to benefit from medical treatment across medical conditions.

However, despite the growing number of studies investigating expectations in different medical conditions, it is difficult to integrate current findings. The heterogeneity with regard to the conceptualization and assessment of patients’ expectations (van Hartingsveld et al., 2010; Bowling et al., 2012; Zywiel et al., 2013) has been considered as a major limitation in several systematic reviews and meta-analyses (Mondloch et al., 2001; Fadyl and McPherson, 2008; Haanstra et al., 2012; Auer et al., 2016a). Some theoretical concepts refer to overlapping aspects of expectations using different terminology, which further complicates the integration of evidence regarding patients’ expectations (Maddux, 2007). Moreover, many studies focus on a single or only a few aspects of expectations, making it difficult to investigate the differential influence of distinct expectation concepts (Haanstra et al., 2015b; Laferton et al., 2015a; Auer et al., 2016b).

Unambiguous terminology, conceptual integration, and standardized assessment are required in order to foster understanding and clinically harness the relationship between expectations and health. The current review has two aims. First, based on a review of current expectation concepts, we aim to provide an integrated model of patients’ expectations in medical treatment. Second, we review the most relevant existing assessment tools and provide recommendations for improving the assessment of expectations with the aim of facilitating more integrative and standardized future research.

## Patients’ Expectations Regarding Medical Treatment: An Overview of Concepts

**FIGURE 1:**
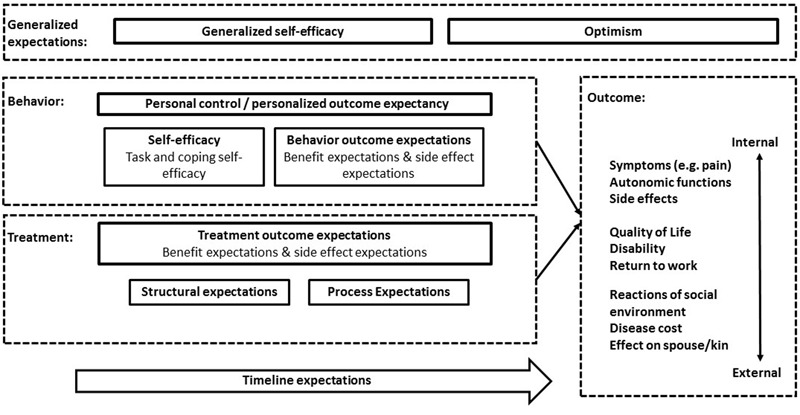
**Schematic illustration of the integrative model of expectations in patients undergoing medical treatment.** Behavior, treatment and outcome related aspects of expectations refer to the specific treatment context. Generalized expectations are independent of the specific treatment context, but might influence specific expectations and treatment outcome. Timeline expectations refer to temporal aspects of the disease, treatment and health behavior, e.g., the course of disease in the context of the treatment.

Expectations are among the most studied constructs in psychological research and have been explicitly or implicitly embedded in many psychological theories (Maddux, 1999). There are many types of expectations in the literature with often ambiguous terminology (Bowling et al., 2012). In the following, theoretical concepts and aspects of patients’ expectations, which are of relevance for health outcomes in medical treatment contexts, are reviewed. They are summarized within an integrative model of expectations of patients undergoing medical treatment (see **Figure 1**) to facilitate an unambiguous and more integrated use of terminology and concepts.

In this manuscript, the term patients’ expectations refers to future-directed beliefs that focus on the incidence or non-incidence of a specific event or experience (Kube et al., 2016). They can manifest as conscious future-directed cognitions, or they may be present without full awareness (e.g., in the case of conditioned learning processes; Kirsch, 2004; Kirsch et al., 2004, 2014). In this sense, expectations are of a predictive nature and need to be distinguished from constructs that have been termed *ideal expectations, value expectations* or *fantasies* (Kravitz, 1996; Oettingen and Mayer, 2002; David et al., 2004; Leung et al., 2009). The latter constructs refer to what a patient would like to happen and are more an expression of hopes or desires than a probabilistic estimation about the future. Ideal expectations or fantasies seem to have opposite effects on health outcomes when compared with patients’ predictive expectations, which empirically confirms the differentiation between the two constructs (Oettingen and Wadden, 1991; Oettingen and Mayer, 2002; Kappes and Oettingen, 2011; Johannessen et al., 2012; Oettingen, 2012).

The following overview of expectation concepts includes social learning and social cognitive theories, the response expectancy theory, the common sense model of illness representation, as well as a short summary of other expectation dimensions. Importantly, our review does not claim to be exhaustive, but rather aims to integrate the most relevant theoretical concepts.

### Social Learning and Social Cognitive Theories

Among the most prominent theoretical backgrounds for the conceptualization of expectations are social learning and social cognitive theories (Bandura, 1986; Maddux, 1999; Schwarzer, 1994), which distinguish two main concepts of expectations: (1) *Behavior outcome expectancies* express the (subjective) likelihood that a specific outcome will follow a given action (e.g., regular exercise will lead to health benefits). These outcomes can be of a physical, social or self-evaluative nature (Bandura, 1997); (2) *self-efficacy* expresses an individual’s expectation of being capable of executing a certain action (e.g., ability to exercise regularly). Self-efficacy can be further distinguished into *task self-efficacy* and *coping self-efficacy* (Kirsch, 1995). While the former expresses the perceived ability to perform a particular behavior (e.g., being able to perform a specific exercise, e.g., jogging), the latter refers to the ability to prevent, control or cope with the demands that might be experienced when performing the behavior (e.g., being able to motivate oneself for regular exercise or being able to tolerate exercise-induced exhaustion). Self-efficacy and behavior outcome expectations play an important role in volitional agentic behavior (Bandura, 2001). However, they do not fully account for the relationship between expectations and non-volitional responses to treatment (Maddux, 1999), such as cardiovascular functions, immune and endocrine functions or pain, as shown by research on the placebo effect (Price et al., 2008; Enck et al., 2013). Non-volitional responses are especially important for expectations regarding medical treatments. Although patient behavior such as medication adherence (Sokol et al., 2005) or a healthy lifestyle (Willett, 2002) plays an important role in medical conditions, in most medical treatments, the patient is largely a responder to external stimuli (e.g., medication, surgical procedures, manual therapy, radiation).

### Response Expectancy Theory

Kirsch’s (1983, 1997) response expectancy theory adds further important aspects of expectations, differentiating between stimulus expectancies and response expectancies. Accordingly, with regard to the outcome that is expected to occur, Kirsch distinguishes between expected external/environmental outcomes (*stimulus expectancies)* and expected non-volitional, internal outcomes (*response expectancies)*. He argues that most theories of expectations are concerned with stimulus expectancies, such as the expectation of money or recognition by others as a result of a certain behavior (Kirsch, 1983). Response expectancies, on the other hand, refer to the expected occurrence of the individual’s non-volitional, internal responses to a certain external stimulus (e.g., the expectation that an analgesic will lead to pain reduction) or to one’s own behavior (e.g., the expectation that a relaxation exercise will reduce subjective stress). Thus, response expectancies cover both aspects of medical treatment: the patient as a passive recipient of medical treatment and the patient’s volitional health-directed behavior. Moreover, expectations regarding non-volitional responses such as change in symptoms or autonomic bodily functions are of outmost importance for patients with medical conditions, as they are often the focus of the disease experience.

### Common Sense Model of Illness Representation

According to the common sense model of illness representation (Leventhal et al., 1980), patients have subjective models about their illness, which comprise interrelated beliefs about the illness and its effect on their lives (Petrie and Weinman, 2012). These beliefs are related to important health outcomes in a broad range of medical conditions (Hagger and Orbell, 2003; Petrie et al., 2007). A patient’s illness perceptions include beliefs about what caused the illness (*causes*), how long it will last (*timeline*), the *consequences* for the patient’s life, which symptoms are attributed to the illness (*identity*), and how the condition can be controlled or cured by the patient’s behavior (*personal control*) or by the treatment (*treatment control*). Although the common sense model does not include expectations as an explicitly denoted construct, expectations are conceptualized as a major underlying component of the different beliefs (Cameron and Leventhal, 2003). For instance, expectations are an inherent part of illness beliefs, including the prediction of future events or experiences, thus referring to timeline, personal control and treatment control as well as (future) consequences. In this regard, the common sense model covers important dimensions of patients’ expectations related to their illness and treatment.

### Additional Dimensions of Expectations

Several other aspects of expectations have been mentioned in the literature (Bowling et al., 2012). *Process* or *structural expectations* (e.g., sequence of steps in a treatment procedure; shape and color of a medication; a physician’s treatment ritual) are an important part of the context in which a treatment takes place, which in turn is a major factor in the placebo effect (Di Blasi et al., 2001; Colloca and Miller, 2011a). Expectations about the structural or process-related aspects of a treatment are likely to influence outcome expectations. For example, expectation effects for the same analgesic are higher when it is applied via a syringe rather than in pill form (de Craen et al., 2000) or when it is openly administered by a physician compared to hidden administration by an automatic device (Price et al., 2008). Similarly, cardiac patients have higher outcome expectations for more invasive procedures (Hirani et al., 2008).

A more self-evident aspect is the *valence* of patients’ expectations. This can be conceptualized either on one dimension, namely expectations of high vs. low *treatment benefit* (e.g., expectation that a treatment will relieve all pain vs. some pain), or on two relatively independent dimensions, namely expectations of treatment benefit and treatment-related *side effects* (e.g., expecting that a treatment will lead to both pain relief and distressing side effects like nausea). Negative expectations about side effects or adverse events can themselves induce the experience of nocebo-related side effects (Barsky et al., 2002; Colloca and Finniss, 2012). Moreover, distinct positive and negative dimensions also apply to behavior outcome expectations (Schwarzer, 1994), e.g., conceptualized as cost and benefit expectations in the *Health Belief Model* (Becker, 1974).

Expectations can further vary in their degree of *specificity* or *generalization*, meaning that they can be held for very specific contexts only (e.g., a specific treatment for a specific medical condition), for several similar contexts (e.g., a specific medical condition or a specific treatment), or ultimately any situation. The most prominent generalized outcome expectation is the concept of *dispositional optimism* (Carver et al., 2010; Hanssen et al., 2013), which has been extensively linked to favorable health outcomes. Notably, dispositional optimism has also been associated with an enhanced placebo response (Geers et al., 2010). In a similar vein, self-efficacy expectations can be context-specific, domain-specific or can ultimately be applied to a broad range of behaviors, as conceptualized in the concept of *generalized self-efficacy* (Schwarzer, 1994; Schwarzer and Jerusalem, 1995).

Other aspects include the *strength of expectations* and their *relation to reality*. The former refers to how strongly a person is convinced of his/her expectation, hence resembling a subjective reality. The latter is a judgment about how realistic an expectation actually is or was. This can only be assessed *post hoc*, or might be estimated based on existing empirical findings or expert judgments.

### Integrative Model of Expectations in Patients Undergoing Medical Treatment

To summarize, several aspects have to be considered for an integrative model of expectations in patients undergoing medical treatment (see **Figure 1**). Expectations can either be related to a patient’s illness- and treatment-related behavior or to the treatment the patient is receiving (Crow et al., 1999; van Hartingsveld et al., 2010). However, contrary to previous conceptualizations (Crow et al., 1999), which considered self-efficacy as the only aspect of expectations regarding patient behavior, one can argue that behavior-related expectations should be divided into self-efficacy and behavior outcome expectations. A patient with high self-efficacy for engaging in regular physical exercise will not start exercising unless he/she also expects exercising to lead to health benefits (behavior outcome expectation). The combination of self-efficacy and behavior outcome expectations has been termed *personalized outcome expectancy* (Kirsch, 1995) or *personal control beliefs* (Cameron and Leventhal, 2003). Treatment-related expectations consist of expectations regarding treatment outcome as well as the structural and process-related aspects of the treatment (Haanstra et al., 2013), which are likely to influence treatment outcome expectations. Both behavioral and treatment outcome expectations can refer to distinguishable expectations of benefits and side effects. Moreover, the expected outcome of a behavior or treatment can be distinguished into the two basic categories described above: (1) expectations of non-volitional, internal changes such as symptoms or autonomic functions, and (2) external expectancies, referring to the expectations of external changes such as reactions of the social environment. Moreover, patients hold expectations about the temporal dimension of their behavior, treatment, disease and the expected outcomes (*timeline expectations*). Finally, it is necessary to consider generalized expectations, such as generalized self-efficacy and generalized outcome expectations (optimism), as these have been shown to influence outcome and are likely to influence specific aspects of expectations in patients undergoing medical treatment (Schwarzer, 1994; Carver et al., 2010).

## Operationalization of Expectations

The proposed model of expectations of patients undergoing medical treatment not only aims to resolve ambiguity on a theoretical level, but also applies to the assessment and therefore the reporting of results on expectation effects. To facilitate the aggregation of evidence on differential aspects of expectations, the model seeks to foster a consistent operationalization and assessment of expectation constructs. In many studies that do not rely on precise terminology and explicit theoretical concepts, these issues can only be detected by inspecting the original items used in the expectation assessment (Kirsch, 1995). The use of the conceptual distinctions of expectations and their precise terminology reviewed in this manuscript should facilitate the resolution of such issues in future research. In the following, examples of instruments assessing expectations in patients undergoing medical treatment are classified in the context of the proposed integrative model of expectations. Subsequently, several issues of the current practice of expectation assessment are pointed out to encourage the advancement of future operationalization.

### Overview of Assessment Instruments According to the Integrative Model of Expectations

Given the aforementioned heterogeneity of assessment instruments, it is beyond the scope of the present work to provide an exhaustive review of assessment instruments for expectations in the medical treatment context. More importantly, in the following paragraph, we will review instruments of relevance to the integrative model of patients’ expectations. **Table 1** identifies the expectation dimensions that are assessed by the outlined instruments.

**Table 1:** Overview of instruments with regard to the aspects of the integrative model of expectations in patients undergoing medical treatment.

Instrument	Expectation construct	Dimensionality	Generic/specific
IPQ-R/B-IPQ (Moss-Morris et al., 2002; Broadbent et al., 2006)	• Personal control (s) • Treatment outcome (s) • Timeline (s) • Consequences (s; if formulated toward the future; see McCarthy et al., 2003; Laferton et al., 2013)	Multi	Generic
FERLHDS (Axelrad, 1982) and C-SPEQ (Holmes et al., 2016)	• Personalized outcome expectancy (i) • Treatment outcome (i) • Process (i) • Timeline (i)	Mixed	Specific
PHES (Leedham et al., 1995)	• Treatment outcome (i) • Timeline (i) • Optimism (i)	Mixed	Generic
SE-ICD and OE-ICD (Dougherty et al., 2007)	• Self-efficacy (s) • Behavior outcome expectations (s)	Multi	Specific
CAS-R (Moser et al., 2009)	• Personalized outcome expectancy (s; “perceived control”)	Single	Specific
LOT-R (Scheier and Carver, 1985)	• Generalized outcome expectancy (s; optimism and pessimism)	Single	Generic
GSE (Schwarzer and Jerusalem, 1995)	• Generalized self-efficacy (s)	Single	Generic
MODEMS (Tashjian et al., 2007)	• Treatment outcome (s)	Single	Specific
NKSSS (Noble et al., 2012)	• Treatment outcome (s)	Single	Specific
PDI-E (Laferton et al., 2013)	• Treatment outcome (s)	Single	Generic
ADL-E (Dohnke et al., 2006)	• Treatment outcome (s)	Single	Generic
PCS-E (Powell et al., 2012)	• Treatment outcome (s)	Single	Generic
CEQ (Devilly and Borkovec, 2000)	• Treatment outcome (s)	Single	Generic
EXPECT-ICD (Habibovic et al., 2014)	• Positive treatment outcome (s); • Negative treatment outcome (s)	Multi	Specific
GASE-EXPECT (von Blanckenburg et al., 2013)	• Negative treatment outcome (s)	Single	Generic
ANP-E (Hüppe et al., 2013)	• Negative treatment outcome (s)	Single	Specific

*(s) = aspect represented by independent scale. (i) = aspect represented by singular item. Dimensionality: Multi = Several expectation dimensions are each assessed by an independent scale; Mixed = Several expectation dimensions are assessed by single items that are subsumed in one scale; Single = Only one expectation dimension is assessed.*

#### Multidimensional Instruments

The instrument that assesses the broadest range of expectation aspects using distinguishable scales is the Revised Illness Perceptions Questionnaire (IPQ-R; Moss-Morris et al., 2002) and its short form (Brief Illness Perceptions Questionnaire; B-IPQ; Broadbent et al., 2006). This very well established instrument offers the possibility to distinguish between treatment control expectations, personal control expectations, timeline expectations and, if reformulated to refer to the future, expected consequences (McCarthy et al., 2003; Laferton et al., 2013), thus satisfying the required multidimensional assessment of expectations.

#### Mixed-Dimensional Instruments

As shown in **Table 1**, most assessment instruments are not specific to a certain concept of the integrative model of expectations, and many of them aggregate items in relation to several dimensions within one expectation score. For instance, the Future Expectations Regarding Life with Heart Disease scale (FERLHDS; Axelrad, 1982) has been used several times in patients with heart disease and has shown acceptable internal consistency as well as construct and predictive validity (Brummett et al., 2004; Chunta, 2009; Barefoot et al., 2011). The measure has recently been adapted for patients undergoing cardiac surgery, again with acceptable reliability and validity (C-SPEQ; Holmes et al., 2016). Both scales use items assessing behavior- and treatment-related expectations with respect to disease-specific and more general expected outcome that are either positively or negatively framed and concern both internal and external outcome expectations. Furthermore, singular items refer to process and to some extent timeline expectations. All 18 items are summed up to form a single expectation score. Additionally, the Positive Health Expectations Scale (PHES; Leedham et al., 1995) has been used in several cardiac surgery populations (Leedham et al., 1995; Sears et al., 2004; Auer et al., 2016b); its internal consistency as well as construct and predictive validity have been confirmed. The scale primarily assesses treatment outcome expectations in relation to more general outcome dimensions such as general physical functioning and quality of life. Additional items ask about motivational aspects and general outlook on life. Again, all items are integrated into a single expectation score.

#### Unidimensional Instruments

Given the impact of social learning theories, self-efficacy has been more frequently operationalized on an explicit theoretical basis compared to most other aspects of patients’ expectations (Bowling et al., 2012). Specific self-efficacy has been assessed in relation to various medical conditions and health behaviors (e.g., Holden, 1991), leading to a large number of specific self-efficacy instruments, for instance for walking (Jenkins and Gortner, 1998), physical exercise (e.g., Schwarzer et al., 2008), nutrition behaviors (Schwarzer and Renner, 2016) or rehabilitation behavior (Waldrop et al., 2001). An exhaustive review of specific self-efficacy instruments is beyond the scope of this manuscript. Only a small number of instruments incorporate both aspects of behavior-related expectations: self-efficacy and behavior outcome expectations. The parallel assessment of both constructs is not indicated if the outcome is largely determined by one’s behavior (Maddux, 1999). However, if this is not the case, it might be valuable to measure personalized outcome expectations or to assess both self-efficacy and behavior outcome expectations. For example, Dougherty et al. (2007) developed a scale that assesses both self-efficacy and behavior outcome expectations in patients undergoing cardioverter defibrillator implantation. Besides the IPQ scales, another instrument assessing the aspect of perceived personal control is the Control Attitudes Scale (CAS; Moser and Dracup, 1995) and its revised form (CAS-R; Moser et al., 2009), which has been psychometrically evaluated in cardiac patients.

Furthermore, several instruments assess generalized expectation constructs. The Life Orientation Test and its revised version (LOT-R; Scheier and Carver, 1985; Scheier et al., 1994), which assess dispositional optimism, constitute a standardized measure that has been extensively evaluated and which further provides population-based norm values (Glaesmer et al., 2012). Moreover, generalized self-efficacy can be assessed with a standardized, psychometrically well-evaluated instrument, the Generalized Self-Efficacy Scale (GSE; Schwarzer and Jerusalem, 1995).

Regarding treatment outcome expectations, a frequent strategy is to adapt instruments or criteria which are commonly used to assess treatment outcome. Following this strategy, some instruments incorporate disease-specific treatment outcome expectations, such as the expectation module of the Musculoskeletal Outcomes Data Evaluation and Management System (MODEMS; Tashjian et al., 2007) or the expectation module of the New Knee Society Scoring System (NKSSS; Noble et al., 2012). Similarly, studies investigating placebo effects have assessed expectations in terms of expected treatment outcome (Bingel et al., 2011; Kirsch et al., 2014).

Other instruments assess treatment outcome expectations by exclusively asking about generic outcome dimensions such as disability, return to work or quality of life. The Pain Disability Index (Tait et al., 1990) has been recently adapted (PDI-E; Laferton et al., 2013) to assess expected disability in seven areas of daily living. So far, it has been used in two independent studies assessing expectations of peripheral arterial disease (Ferrari et al., 2015) or heart surgery (Rief et al., 2017). It was shown to be have good internal consistency (Laferton et al., 2015b) and construct validity (Laferton et al., 2015a). In a similar fashion, Dohnke et al. (2006) assessed expectations for activities of daily living (ADL-E) in hip joint replacement rehabilitation patients. Powell et al. (2012) assessed expectations by adapting the SF-36 physical functioning quality of life component score (PCS-E), although both of the aforementioned studies failed to report the psychometric evaluation of the scales. Another generic way to assess patients’ expectations is to ask about their perceived likelihood of return to work (Fadyl and McPherson, 2008), which is highly relevant for many patients. Finally, the Credibility Expectancy Questionnaire (CEQ; Devilly and Borkovec, 2000) is an evaluated and frequently used instrument to assess patients’ perceived treatment credibility and treatment outcome expectations on a generic level. Originally, the CEQ was developed for application within psychotherapeutic treatment, but it can be easily adapted for the medical treatment context (e.g., Haanstra et al., 2015a,b).

Few instruments exist for the specific assessment of negative outcome or side-effect expectations. The EXPECT-ICD (Habibovic et al., 2014) assesses positive and negative treatment outcome expectations of patients undergoing cardioverter defibrillator device implantation. The scale includes items assessing both disease-specific outcome dimensions and more generalized outcome dimensions such as physical functioning and quality of life. Moreover, some instruments specifically assess side-effect expectations for pharmacological treatment. The General Assessment of Side Effects Scale (Rief et al., 2011) assesses the most common medication side effects and has recently been adapted for the assessment of expectations about side effects of breast cancer patients undergoing endocrine therapy (GASE-EXPECT; von Blanckenburg et al., 2013). It has shown good initial internal consistency and validity (Heisig et al., 2015; Nestoriuc et al., 2016) and can be adapted to incorporate medication-specific symptoms. In a similar vein, Hüppe et al. (2013) assessed expectations for general anesthesia-related side effects by adapting the Anaesthesiological Questionnaire (ANP-E; Hüppe et al., 2003) for the measurement of side effects. Moreover, several measurement instruments have been developed based on the common sense model of illness representation. These instruments incorporate treatment concerns, which combine expectations about side effects with more general aspects of worrying in the context of treatment. The subscale “concerns” of the Beliefs about Medicines Questionnaire (Horne et al., 1999) incorporates expectations about negative effects of medications. Similar instruments have also been developed to assess concerns about surgery (Francis et al., 2009) or heart disease treatment (Hirani et al., 2008).

In sum, although some standardized measurements have been developed to assess different aspects of expectations, very few studies have examined the extent to which these different measures conceptually overlap (e.g., Haanstra et al., 2015b; Laferton et al., 2015a; Auer et al., 2016b; Heisig et al., 2016). Despite this variety of assessment instruments, the current practice of assessing patients’ expectations in the medical treatment context can be further improved. In the following, we provide recommendations for improving the future assessment of expectations in patients undergoing medical treatment.

### Recommendations for Improving the Assessment of Expectations in Patients Undergoing Medical Treatment

#### Standardized Assessment

Several reviews concluded that there is a lack of standardized assessment of medical patients’ expectations (Fadyl and McPherson, 2008; Haanstra et al., 2012; Auer et al., 2016a). Besides lacking conceptual standardization as discussed above, many instruments were developed and used for only one investigation, often without providing a rationale for development or data on psychometric evaluation (van Hartingsveld et al., 2010; Bowling et al., 2012; Zywiel et al., 2013). This is a major issue, as without knowledge about reliability and validity, the evidence collected using such an instrument is subject to major limitations. To gather more credible evidence, measurement instruments need to be developed based on a transparent rationale. Possible strategies may include theory-guided development, qualitative research on patients’ expectations, expert focus groups or the adaptation of well-developed patient-reported outcome tools. Further, the dimensionality of the measurement tool not only needs to be developed in an exploratory manner, but also needs to be tested in a confirmatory manner in independent samples. Moreover, reliability, construct validity and predictive validity need to be confirmed across several studies.

#### Multidimensional Assessment

A further issue is the lack of multidimensionality. Many studies merely assess one aspect of expectations (e.g., behavior- vs. treatment-related expectations; van Hartingsveld et al., 2010; Zywiel et al., 2013). If one wishes to assess the expectation effects in relation to a single application of an analgesic (e.g., in an experimental investigation of placebo effects), the assessment of treatment-related expectations might cover most of the relevant expectations in that context. The same might apply to studies investigating expectation effects related to patient behavior in the absence of any treatment. However, this hinders the collection of integrative evidence regarding the predictive value of distinct aspects of expectations in medical conditions (see also Auer et al., 2016a,b). This is also problematic for clinical practice, as for the majority of patients with medical conditions, several aspects of expectations appear to be important (e.g., expectations about treatment efficacy, personal control over as well as consequences of a particular disease; Haanstra et al., 2013). Measuring only one aspect does not cover the whole picture. Similarly, if several aspects of expectations were assessed at the same time, but were not distinguished by separate (sub-)scales of the instrument, this would impede knowledge about the differential role of certain aspects of expectations. Therefore, the parallel application of instruments measuring different aspects of expectations or the use of an instrument distinguishing certain aspects of expectations is essential. The parallel assessment of the dimensions listed in the following paragraphs should be especially considered when assessing medical patients’ expectations.

As mentioned above, in most medical treatment contexts, both the patients’ illness- and treatment-related behavior and the treatment itself are important factors for treatment success (Crow et al., 1999; van Hartingsveld et al., 2010). Therefore, both treatment- and behavior-related expectations are likely to influence health outcomes. Yet, very few instruments incorporate separate scales for both aspects of expectations (see **Table 1**) and only a small number of studies use separate instruments to measure both treatment- and behavior-related expectations. For example, in a review of measurements for expectations of patients with musculoskeletal disorders (van Hartingsveld et al., 2010), only one out of 24 studies attempted to measure both features. Assessing these aspects of expectations separately could facilitate a more differential understanding of expectation effects and would help to inform the design of interventions targeted at patients’ expectations in medical conditions. Of the instruments described above, only the IPQ-R and the B-IPQ offer the possibility to assess several aspects of expectations on distinct scales. An alternative option would be the parallel use of validated instruments for both treatment-related expectations and behavior-related expectations.

Another neglected aspect is the assessment of patients’ expectations regarding adverse effects or side effects of treatment and health behavior. As described above, few instruments assess side-effect expectations. While some measurement instruments incorporate both items about positive and negative outcome expectations (see **Table 1**), they are often subsumed in one scale (by reverse-coding items with negative expectations). However, expectations about positive and negative effects do not necessarily belong in one dimension. As an example, a study assessing expectations of patients who had undergone implantable cardioverter defibrillator implantation (Habibovic et al., 2014) revealed two distinguishable factors of positive and negative expectations, of which only negative expectations predicted higher levels of anxiety, depression and concerns at 3-month follow-up. Distinguishing between expectations of benefits and adverse effects might be especially valuable if they affect different dimensions of outcome and different timeframes. For instance, a patient undergoing coronary artery bypass graft surgery might expect a benefit in reducing shortness of breath in the long term, but might also expect pain in the short-term post-surgery period. In such a scenario, summing up the two aspects of expectations would be counterintuitive. While the majority of existing measurement instruments assess benefit expectation, only a small number have been used to separately assess side-effect expectations. Moreover, we are not aware of any instrument assessing expected adverse effects of health behaviors. Assessing these side effects might explain additional variance in patients engaging or not engaging in health-related behavior.

Further aspects that are underrepresented in studies assessing expectations are stimulus/external outcome expectations, process/structural expectations and timeline expectations. As mentioned above, outcome expectations can be related to internal response expectations or to expectations regarding external effects of illness and treatment, such as financial consequences or consequences affecting significant others. The majority of measurement instruments, however, focus on response expectancies. External factors, such as the consequences of treatment on a spouse, can be of significant importance in patients undergoing medical treatment. Therefore, assessing such external outcome expectations might further complete the picture of patients’ expectations.

Expectations about the process and the structure of treatments are more difficult to assess in complex treatments, which might be a reason why few instruments attempt to capture these aspects. Relevantly, evidence from qualitative research shows that patients do hold quite specific process- and structure-related expectations (Haanstra et al., 2013). As these aspects are related to treatment outcome (see above), it would be worthwhile to assess them more systematically in patients in medical care. Finally, expectations about the temporal course of a disease have been shown to be predictive of several health outcomes across medical conditions (Broadbent et al., 2015). So far, this aspect of expectations has most often been operationalized explicitly in studies using the IPQ-R and B-IPQ. Given their predictive value, future studies should consider assessing expectations regarding temporal course more often.

#### Specific vs. Generalized Assessment of Expectations

As expectations are to a substantial extent situation-specific, the majority of instruments assess expectations for a specific treatment of a particular medical condition. As a result, even within one single category of medical conditions (e.g., musculoskeletal; van Hartingsveld et al., 2010; Zywiel et al., 2013), a high heterogeneity of expectation assessment can be found. This makes it difficult to compare the differential impact of certain expectations across different treatments and illnesses.

Likewise, with regard to the assessment of outcome expectations too, instruments differ in their specificity, assessing expectations about rather disease-specific symptoms or functions (e.g., degree of joint rotation, sexual functioning), generic symptoms (e.g., pain, sleep), broadly applicable concepts like disability, quality of life or return to work, or trait-like generalized outcome expectations (e.g., optimism, hope). Many instruments assess expectations on a disease- or treatment-specific level, meaning that they are not applicable to other conditions. Thus, expectation effects cannot be compared across conditions. The assessment of generalized outcome expectations like optimism is possible for any condition. However, this does not provide any insight into the patient’s expectations while receiving medical treatment, as such instruments capture expectations on a very abstract level, with no specific reference to the treatment context. A solution to balance these two goals might be to measure expectations regarding expected disability, quality of life, or return to work (see **Table 1**). In contrast to disease-specific outcome instruments, the assessment of these kinds of expectations would be applicable to any disease or treatment. At the same time, such an assessment could still ask about concrete entities that are relevant for the patient’s specific illness and treatment experience, as opposed to assessing outcome expectations on a very abstract basis, as is the case with optimism and similar concepts.

#### Additional Aspects to Consider

In addition to the aforementioned points, the timing of the assessment should be taken into consideration when assessing patients’ expectations: Expectations have been assessed before, shortly after or at recovery/follow-up of a treatment or diagnostic test (Zywiel et al., 2013). Most studies have assessed expectations prior to the treatment or the diagnostic procedure (van Hartingsveld et al., 2010), which seems logical since these are salient events that are likely to trigger expectations. Presumably, expectations might be influenced by the course of treatment or diagnostic procedure. However, the effects of different assessment timing remain unclear, as they have rarely been investigated systematically (e.g., van den Akker-Scheek et al., 2007). Therefore, to investigate the temporal course of patients’ expectations and the influencing factors, they should be assessed at multiple time points in the course of a treatment or a diagnostic procedure (Kamper et al., 2015). Moreover, assessing expectations on multiple occasions (before, during, and after a procedure) might foster knowledge about the stability of expectations. Additionally, researchers should always consider the burden of assessment with regard to the patient’s condition. However, as most expectation scales are brief and intuitive, this should not be a problem in most cases. Finally, although the main focus of this review was on patients’ expectations, the expectations of healthcare providers/physicians may also play a critical role for treatment outcomes. Studies examining the relevance of physicians’ expectations are scarce, although they have been shown to be related to treatment outcomes at least in some studies (e.g., Gracely et al., 1985; Galer et al., 1997; Witt et al., 2012). Further, there is evidence that if physicians communicate their high expectations to their patients, the patients’ expectations are increased (Crow et al., 1999; Verheul et al., 2010). Certainly, it could be valuable to assess physicians’ expectations and their impact on treatment outcomes in order to further explore the role of expectations in the medical treatment context. In particular, future studies should endeavor to elucidate the relationship between physicians’ expectations and patients’ expectations. The latter may mediate the effects of the former on treatment outcomes.

## Conclusion

Patients’ expectations in the context of medical treatment constitute a promising area of research, as growing evidence suggests that they have an influence on health outcomes across a variety of medical conditions. However, the aggregation of evidence is complicated by an inconsistent and disintegrated application of expectation constructs and the heterogeneity of assessment strategies. Within this review, we outlined an integrative model of expectations that aims to facilitate the consistent use of expectation constructs and more theory-driven standardized assessment strategies. In particular, the application of standardized, psychometrically evaluated measures, assessing multidimensional aspects of patients’ expectations that are applicable across various medical treatments has the potential to generate a more comprehensive understanding of expectation effects in medical treatments. Future research should overcome the current obstacles in assessing expectations as outlined above. Moreover, more research is needed on the interrelation of different expectation aspects as well as on factors influencing patients’ expectations of illness and treatment in clinical populations. Most studies investigating this question in medical patients have done so cross-sectionally (e.g., Scott et al., 2012; Laferton et al., 2015a). Prospective studies are warranted to gain a better understanding of the direction of influencing variables (e.g., demographic, medical, and psychosocial).

This might ultimately facilitate interventions aiming to influence patients’ expectations in order to improve health outcomes. Patients’ expectations can be effectively modulated by verbally suggesting that treatment is beneficial (Bingel et al., 2011; Kam-Hansen et al., 2014), using an empathetic interaction style (Kaptchuk et al., 2008), or discussing patients’ treatment beliefs and concepts (Laferton et al., 2015b). Recently, several clinical intervention studies have shown that patients’ expectations can be optimized via brief psychological interventions and that these interventions ultimately lead to improved health outcomes (Broadbent et al., 2009; von Blanckenburg et al., 2013, 2015; Rief et al., 2017). The application of theory guided frameworks, such as the ViolEx-model on expectation development, expectation maintenance, and expectation change proposed by Rief and Petrie (2016), might further help to refine such interventions. In this regard, an integrated understanding and assessment of patients’ expectations is the first step toward improved health care across medical conditions.

## Author Contributions

JL, TK, CA, SS, and MS-M: Substantial contributions to the conception and design of the manuscript; drafting the work or revising it critically for important intellectual content; final approval of the version to be published; agreement to be accountable for all aspects of the work in ensuring that questions related to the accuracy or integrity of any part of the work are appropriately investigated and resolved.

## Conflict of Interest Statement

The authors declare that the research was conducted in the absence of any commercial or financial relationships that could be construed as a potential conflict of interest.
